# Function after spinal treatment, exercise and rehabilitation (FASTER): improving the functional outcome of spinal surgery

**DOI:** 10.1186/1471-2474-11-17

**Published:** 2010-01-26

**Authors:** AH McGregor, CJ Doré, TP Morris, S Morris, K Jamrozik

**Affiliations:** 1 Surgery & Cancer, Faculty of Medicine, Imperial College London, Charing Cross Hospital Campus, London W6 8RP, UK; 2 MRC Clinical Trials Unit, 222 Euston Road, London, NW1 2DA, UK; 3 Department of Epidemiology and Public Health, University College London, London, WC1E 6BT, UK; 4 School of Population Health and Clinical Practice Office, North Terrace, University of Adelaide, SA 5005, Australia

## Abstract

**Background:**

The life-time incidence of low back pain is high and diagnoses of spinal stenosis and disc prolapse are increasing. Consequently, there is a steady rise in surgical interventions for these conditions. Current evidence suggests that while the success of surgery is incomplete, it is superior to conservative interventions. A recent survey indicates that there are large differences in the type and intensity of rehabilitation, if any, provided after spinal surgery as well as in the restrictions and advice given to patients in the post-operative period.

This trial will test the hypothesis that functional outcome following two common spinal operations can be improved by a programme of post-operative rehabilitation that combines professional support and advice with graded active exercise and/or an educational booklet based on evidence-based messages and advice.

**Methods/Design:**

The study design is a multi-centre, factorial, randomised controlled trial with patients stratified by surgeon and operative procedure. The trial will compare the effectiveness and cost-effectiveness of a rehabilitation programme and an education booklet for the postoperative management of patients undergoing discectomy or lateral nerve root decompression, each compared with "usual care"using a 2 × 2 factorial design. The trial will create 4 sub-groups; rehabilitation-only, booklet-only, rehabilitation-plus-booklet, and usual care only. The trial aims to recruit 344 patients, which equates to 86 patients in each of the four sub-groups. All patients will be assessed for functional ability (through the Oswestry Disability Index - a disease specific functional questionnaire), pain (using visual analogue scales), and satisfaction pre-operatively and then at 6 weeks, 3, 6 and 9 months and 1 year post-operatively. This will be complemented by a formal analysis of cost-effectiveness.

**Discussion:**

This trial will determine whether the outcome of spinal surgery can be enhanced by either a post-operative rehabilitation programme or an evidence-based advice booklet or a combination of the two and as such will contribute to our knowledge on how to manage spinal surgery patients in the post-operative period.

**Trial Registration:**

Current controlled trials ISRCTN46782945

UK CRN ID: 2670

## Background

Currently we are approaching the end of the Bone & Joint Decade, which at its onset identified low back pain (LBP) as one of its main focuses [1]. Whilst LBP is not a life threatening condition it does affect a large proportion of the population with a point prevalence of between 12-35% and a lifetime prevalence of 49-80%[2]. It is therefore not surprising that it is the second leading cause of sick leave [1], impacting on health care utilisation and contributing to disability and work loss. As such it is one of the most costly health problems facing society. Maniadakis and Gray [2] estimated that there are 3.1 million adults in the UK suffering with LBP each year, costing the UK over £9 billion per annum. Whilst the prevalence of back pain is high, a survey in 1994 by the Office of Population Censuses and Surveys indicated that only 10% of affected individuals attend an NHS outpatient clinic and only 0.5% undergo any surgical intervention[3]. Nevertheless, inpatient treatment, of which surgery is a key component, forms the largest single element of overall expense to the NHS related to back pain[4], possibly as high as 30% of the overall direct cost of back pain[5].

Two of the commonest surgical procedures performed on the spine are discectomies for herniated discs[6,7] and nerve root decompression for spinal stenosis[8]. The rates of both of these interventions are rising, particularly with respect to decompression surgery for spinal stenosis which has been attributed to a growing ageing population [8,9]. The existing literature is sufficiently supportive of the operative management of nerve root stenosis and disc protrusion as to preclude a new trial comparing surgical versus conservative treatment of these conditions [10-16]. However, the success of these surgical procedures and patient satisfaction with the outcome, are variable. In decompression surgery for spinal stenosis, success rates have ranged from 58-69% [7,17,18], with patient satisfaction ranging from 70-81% [9,13,19]. McGregor & Hughes [19] found much lower levels of patient satisfaction which was attributed to unrealistic expectations of surgery. This contrasts with Yee et al[9] who suggested that 81% of patient expectations were met by surgery - however, Yee provided patients with detailed pre-operative information. The approach used to assess satisfaction and type of outcome measures used may also contribute to this variation in findings. It has been suggested that whilst leg pain improves following decompressive surgery, functional improvements are less marked[18,20] and this in turn may impact on quality of life [21], so whilst one outcome is deemed successful others are less so. This suggests that there is scope for improvement, particularly with respect to functional outcome.

Discectomy for disc prolapse has higher success rates, ranging from 65-90%[9,22,23], but residual back and leg pain and recurrent herniation remain the major post-operative problem in lumbar disc surgery [21]. Indeed Soldberg et al [22] reported that 4% of patients got worse after surgery, and Yorimitsu et al [24] indicated that up to 10% had significantly more leg pain and a further 10% had significantly more back pain. In terms of function, Yorimitsu et al [24] noted that only 40% of patients returned to pre-sciatica levels of recreational activity, which concords with the work of Thomas et al [25] which suggested that postoperatively discectomy patients health related quality of life scores remain lower than in the normative population.

The upshot is that more could be done to improve functional outcome and quality of life following both types of surgery. Several approaches have been taken to improve these outcomes including optimising patient selection [1,26,27], refining and exploring new surgical techniques [1,28-31], and lastly improving post-operative care and management [32-34]. In May 2003 a workshop comprising surgeons (both orthopaedic and neurosurgical), physiotherapists, statisticians, economists and epidemiologists was convened to discuss these three different approaches and their relative benefits. The conclusion of this meeting was that there was a paucity of knowledge with respect to post-operative management and that addressing this area may provide significant health impact for this patient population. It was agreed that an important first step was to establish current practice with respect to post-operative management following spinal surgery.

Consequently a national survey of practice in the UK was performed. This revealed wide variation in recommendations to patients by surgeons with respect to activity levels and return to work following surgery [35]. Only 35% of surgeons provided written post-operative instructions; there was limited referral to physiotherapy, with only 45% referring patients to a physiotherapist for an average of 1.8 sessions of treatment; and 18% of surgeons advocated the use of a corset post-operatively, with others restricting sitting or encouraging bed rest. Other studies have also noted marked differences between surgeons in recommendations regarding return to work and lifting [1,36-38]. In summary, although individual surgeons may be certain of their practice, the overall variation in post-operative management suggests uncertainty and the literature reveals limited evidence for current practices.

With the improvements in pain associated with minimal improvements in function [20,24], there is an increasing body of evidence suggesting a need for rehabilitation, with evidence of muscle dysfunction in back pain patients[1,39,40], with further deterioration and damage to the muscles as a result of surgery [41,42]. However, while there appears to be a need for some form of post-operative rehabilitation, our survey suggests that uptake is currently poor in the UK, with many surgeons providing simple advice only [35]. A review [43] identified thirteen controlled trials that compared an active rehabilitation programme with standard post-operative care in patients undergoing spinal surgery. These studies suggested that a vigorous post-operative exercise regime led to a more rapid return to work. However, most of these trials were small (a mean of 72 participants, range 12-212) and the measures of outcome limited. In addition, the statistical analysis was inappropriate in some instances, with emphasis on longitudinal comparisons within trial groups rather than between-group comparisons at follow-up. Since this review a number of further randomised and cohort studies have been performed comparing a range of interventions from self directed stretching and stabilisation exercises [44], behavioural graded activities [1,33,45], neuromuscular training [46], stabilisation classes and mixed therapies [47] and home educated and self management interventions [33,48]. Again many of these studies were underpowered, with many comparing interventions such as exercise with education rather than using the limited post-operative care detailed in the recent UK survey [35] as the comparator. Consequently although differences between treatment arms were noted, it is not clear what their real impact would be with respect to usual care, and often these differences did not persist at long term review which may be related to the low power.

As a consequence there is a range of different practices with respect to post-operative management with limited scientific basis for these interventions. This would suggest that further large rigorously-designed and well-executed randomised trials are required. Using the information obtained from both the survey and literature review such a study would need to consider the use of educational material, some form of rehabilitation, and/or a combination of both and "usual care". To design such a trial however, necessitates the availability of both a standard rehabilitation programme and appropriate education material. Considering educational material initially, a review of the literature, internet and utilising the information from the survey [35] revealed that there was no standard practice and a wide range in the accuracy and extent of information provided. Thus, prior to commencing a trial appropriate educational material had to be developed. Based on the success of "The Back Book" [49,50], it was felt that a booklet may be an appropriate forum to deliver such information, and that making the content evidence-based would help address the general confusion in the publically available information to patients. Therefore, a large literature review into post-operative management was instigated and this was used to synthesise key messages for a post operative educational booklet which was subsequently evolved, evaluated and published [34,51].

The next step was to develop a post-operative intervention that was acceptable to both surgeons and patients. It was not possible to base this on current evidence as this was lacking, with respect to content and timing. At the initial workshop to develop this study, the surgeons were reluctant to discharge their patient directly into a rehabilitation class and the general consensus was that any form of rehabilitation class should start no sooner than 6 weeks post-operatively. Previous post-operative classes had either focused on stretching, resistance training, spine stabilisation exercises or mixed physiotherapy techniques [44,46,47,52]. With little agreement from these studies on what the key exercise content should be and a lack of evidence in the scientific literature the focus of the classes was to get the patient active, and it was decided to base the content on the style of Frost et al[53] Back to Fitness programme, but to extend these twice weekly classes over 6 weeks rather than 4. Based on the success of the Back Café concept described by Christensen et al [54] where patients benefited from meeting and discussing their surgery and progress with other patients, it was decided to include a discussion forum in the rehabilitation classes and for the classes to be of a rolling nature i.e. classes run continuously with patients joining the classes at their own allocated time rather than waiting for a new batch of classes to start. This facilitates patients at different stages of the post-operative journey sharing their experiences with others in the class. To incorporate all of these issues each class will last a maximum of one hour.

With the interventions in place it was possible to design an appropriate trial, the aim of which was to evaluate via a factorial randomised controlled trial, the benefits of a rehabilitation programme and an education booklet for the postoperative management of patients undergoing discectomy or lateral nerve root decompression, each compared with "usual care". The primary aim of this study is therefore, to determine if the long-term functional outcome of spinal surgery and patient satisfaction can be improved via either a systematic programme of post-operative rehabilitation or an educational booklet, and whether a combination of both is even more effective. A secondary objective is to assess whether or not such approaches are cost-effective.

## Methods/Design

### Trial Design

Function after spinal treatment, exercise and rehabilitation (FASTER) is a multi-centre, factorial, randomised, parallel group controlled trial using validated measures of outcome and with a parallel economic analysis. It involves recruiting and consenting patients with symptoms, signs, and radiological findings of either lateral nerve root compression or disc prolapse scheduled for surgery who will be randomised using a 2 × 2 factorial design to receive:

• Factor 1 - either a six-week programme of post-operative rehabilitation or the relevant surgeon's usual postoperative care, which includes clinical review and perhaps some very limited physiotherapy:

• Factor 2 - either an educational booklet ("Your Back Operation" see below), or the surgeon's usual advice.

**Figure 1:**
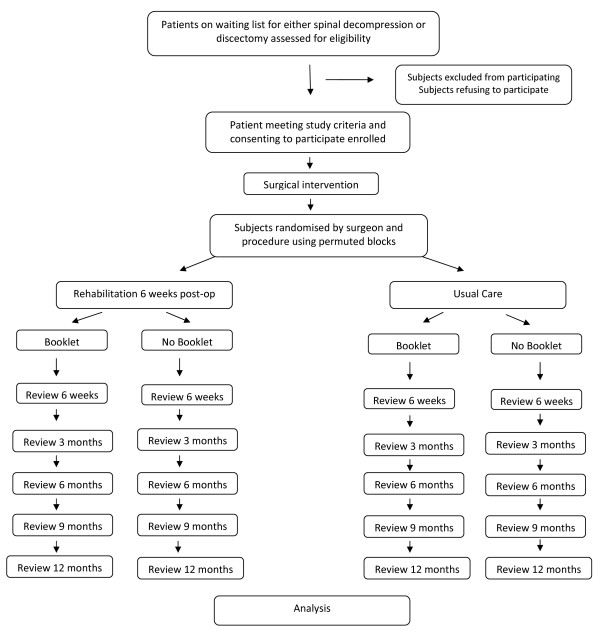
**Flowchart overview of the FASTER study**.

This will create 4 groups; rehabilitation-only, booklet-only, rehabilitation-plus-booklet, and usual care only. Allocation to a group will be stratified by surgeon and surgical procedure and allocation will use random permuted blocks within strata. This ensures that each participating surgeon and both surgical procedures will have approximately equal numbers of patients allocated to the 4 groups. Treatment allocation will be concealed to avoid selection bias during recruitment. All patients will be assessed for functional ability, pain, and satisfaction pre-operatively and then at 6 weeks, 3, 6 and 9 months and 1 year post-operatively. This will be complemented by a formal analysis of cost-effectiveness. A summary is provided in figure 1.

### Interventions

#### Surgical Intervention

All participants will undergo spinal surgery according to their surgeon's routine practice for that condition (i.e. either lateral or central root canal decompression or discectomy). The details of the surgical procedure performed (e.g. open versus micro-discectomy, single versus multiple levels) will be recorded as potential determinants of outcome, and a copy of the operation notes will be obtained for all patients.

#### Rehabilitation Programme

Patients randomised to the rehabilitation arms of the trial will commence the programme 6 to 8 weeks following surgery. The programme will run for 6 weeks with patients attending for 1 hour twice a week. The classes will be run by an experienced physiotherapist, who will encourage patients individually to progress at their own pace. This structure allows new patients to join the programme at any time rather than in 'batches'. There will be a maximum of ten patients per class, located at the hospital where the surgery was performed.

The classes will be standardised using the principles outlined by Frost et al [53] and will include: general aerobic fitness work; stretching; stability exercises; strengthening and endurance training for the back, abdominal and leg muscles; ergonomic training; advice on lifting and setting targets; and self-motivation. Participants will be asked to undertake shorter (30 minute) sessions of exercise at home. The classes will have a cognitive therapy basis, and will include a group discussion/open session at the end of each class where patients can discuss any problems, worries and concerns they have with each other and the physiotherapist. Compliance with classes will be recorded on the basis of attendance, and a subgroup analysis will be conducted by proportion of classes attended. In addition the classes will be reviewed regularly to ensure a uniform quality and content.

#### Educational Booklet

Patients randomised to the booklet arms of the trial will receive a copy of "Your Back operation"[55] on discharge from hospital. This is an evidence-based booklet designed for patients having surgery for either disc prolapse or spinal decompression. Further details on the construction and content of the booklet have been previously reported [34].

#### Usual care

Patients randomised to the usual care control group will be managed according to the relevant surgeon's usual practice. The post-operative regimes of each surgeon will be documented and patients will be questioned via a self-completed questionnaire regarding any interventions received or advice sought.

### Outcome measures

The primary outcome measure is the Oswestry Disability Index (ODI, version 2.1), a disease-specific patient-completed questionnaire documenting function[56] of known validity and reliability [57], measured at the one year follow-up visit. This will be complemented by a series of secondary outcome measures. At baseline (the pre-operative assessment) and each follow-up point, simple 10 cm visual analogue scales (VAS) will be used to record least and usual back and leg pain, from which average values will be derived [58]. Measures of patient satisfaction will focus on the patient's satisfaction with the actual procedure undertaken and its outcome as opposed to satisfaction with the hospital's facilities, staff, etc. This will be assessed directly using a 5 point Likert scale ranging from 'complete improvement' to 'significant deterioration', and indirectly by comparing actual outcome with expected outcome. Expected outcome for pain and function will be assessed pre-operatively and at 6 weeks post-operation using 10 cm VAS, with subsequent satisfaction relating to each of these measures being assessed using similar VAS ranging from 'completely satisfied' to 'completely dissatisfied'[19]. Participants in the trial will be asked to complete the HADS [59], a well validated instrument for the assessment of anxiety and depression among patients in non-psychiatric hospital clinics at each assessment point. This will allow us to take pre-operative psychological status into account as a factor potentially affecting the outcome of surgery. In similar fashion, we will use the physical activity component of the Fear Avoidance Beliefs Questionnaire developed by Waddell et al[55] to take perceptions and expectations into account as possible influences on functional outcome. Information will also be collected on the timing and extent of any return to work, plus the nature of the participant's occupation, and on the timing and nature of any re-operations.

For the economic analysis we will collect data on costs and health-related quality of life (HRQOL) from patients in the trial. Costs will be collected from the perspective of the NHS, personal and social services (PSS) and patients and families. We will include costs for the following components where they are attributable to back pain: post-surgery rehabilitation; education booklet; hospital contacts (inpatient, day case, outpatient); primary care contacts (general practitioner, practice nurse, community nurse); and medications. We will distinguish between costs incurred by the NHS and by patients and families. Resource use data for each cost component will be obtained from patient diaries completed prospectively and collected at each post-operative review. These data will be combined with unit cost data from published national sources [60-62] to compute the costs for each patient in the trial. HRQOL will be measured using the EQ-5D, a validated, global measure of quality of life [63], which will also be measured at every post-operative review.

### Trial Sample

This trial has been approved by Hammersmith and Queen Charlotte's & Chelsea Hospitals Research Ethics Committee, with site approval for Ravenscourt Park Hospital, St Mary's Hospital, St Thomas' Hospital, Royal Free Hospital, Heatherwood and Wexham Park Hospital, and Charing Cross Hospital.

Eligible patients include those currently on the waiting list for spinal surgery with either (a) signs, symptoms and radiological evidence of lateral nerve root compression, that is, patients presenting with radicular pain with an associated neurological deficit or with neurogenic claudication (pain in the buttock, thigh or leg that improves with rest), or (b) lumbar disc prolapse, that is, patients with root symptoms and signs and MRI confirmation of lumbar disc herniation. Patients with any of the following will be excluded from participation in the trial; any condition where either the intervention or the rehabilitation may have an adverse effect on the individual; previous spinal surgery; spinal surgery where a fusion procedure is planned due to the unknown hazards of the activity programme for this type of surgery; pregnant women; inadequate ability to complete the trial assessment forms; any patient who is unable to attend the rehabilitation or the reviews or who is unsuitable for rehabilitation classes.

Written informed consent will be obtained from all patients and baseline assessment completed prior to surgery and randomisation into the trial. Patients will be notified of their randomisation following surgery. Those allocated to either the booklet-only group or the rehabilitation-plus-booklet group, will get the "Your back operation" on discharge (see details below).

The trial aims to recruit 344 patients into the trial with 86 patients in each of the 4 sub-groups. This figure was based on being able to detect a 20% relative improvement in the Oswestry Disability Index (version 2.1). We anticipate a mean ODI of 40 in the control group and 32 in the exercise group at 1 year, to have a 90% chance of detecting a between-group difference of 8 points on the ODI and declaring it statistically significant using a two-sided alpha= 0.05, requires a total of 344 patients. This calculation assumes a standard deviation of 20 in each group and allows for loss to follow-up of 23%, as found in earlier descriptive work [20].

### Proposed Analyses

Analyses will begin with a descriptive comparison of the trial groups before surgery to confirm that randomisation has produced groups that are balanced with respect to known confounders. This will include age, sex, type of surgery, ethnic background, marital status, body mass index, occupation type, work status and smoking status. Pre-operative scores on the primary and secondary outcomes will also be presented by group.

Full functional recovery from back surgery can be protracted, so the primary outcome of interest is the between-group difference in score on the ODI at one-year follow-up, based on intention-to-treat (modified to exclude patients recruited in error). Missing outcome data are expected because of this timeline. Multiple imputation under missing at random [64] will be used for patients with some follow-up but unmeasured one-year outcomes.

Groups will be compared via analysis of covariance, with adjustment for stratifying block using a linear mixed model. If assumptions appear violated, some transformation of the outcome will be taken such that assumptions appear sensible. If this fails, a non-parametric test will be used.

Parallel analyses will be performed for booklet vs. no-booklet and rehabilitation vs. no-rehabilitation, with the sample-size calculation given above applying to each of these. These calculations will be followed by an analysis which tests for an interaction of effect of the two interventions on the primary outcome.

We will check whether the same pattern of outcomes is apparent for patients undergoing lateral nerve root decompression and those undergoing discectomy, and for other subgroups defined by baseline body mass index, baseline HADS score, and single versus multiple anatomic levels of surgery. In addition to the analyses at one year, we will also compare the groups at 6 weeks (beginning of rehabilitation programme in the intervention group) and 3, 6, and 9 months.

The economic analysis will conform to recommended national guidelines [65]. We will undertake two analyses, a short-run analysis and a long-run analysis. In the short-run analysis the time horizon will be one year post-surgery and the analysis will be based on the data collected in the trial. In the long-run analysis we will extrapolate beyond the end of the trial using a de novo economic model to measure lifetime costs and benefits. Cost-effectiveness will be calculated as (1) the incremental cost per unit of improvement in functional outcome at one year, measured in terms of the primary outcome, (2) the incremental cost per quality-adjusted life year (QALY) gained at one year, and (3) the incremental cost per QALY gained over the lifetime. These measures summarise the extra costs and extra benefits of the interventions and can be used to assess whether or not they represent good value for money to the NHS. The economic analysis will include comprehensive deterministic and probabilistic sensitivity analyses to identify the areas of uncertainty in our analysis and the likely impact this will have on the results. We will also undertake a budget impact analysis to assess what the likely financial impact would be to the NHS if the interventions were rolled out nationally.

### Interim analysis

As active rehabilitation may potentially produce a worse outcome, there will be an interim analysis once a third of the target number of patients have reached 6 month follow-up, this would allow for 60 patients per group for the main effect of each factor. At this analysis, an independent Data Monitoring Committee (DMC) will apply stopping rules of p < 0.01 for harm, and p < 0.001 for benefit associated with each intervention. We are assuming that there is no interaction between the rehabilitation and booklet however we have requested that the DMC inform us if it becomes apparent that this is not the case. If the DMC recommend stopping recruitment to one of the trial factors only, recruitment and randomisation regarding the other factor will continue but the intervention to which the stopping rule has been applied will be applied (or withheld) for all patients subsequently randomised. For example, if an interim analysis demonstrates a significant beneficial effect of rehabilitation (based on the pre-specified stopping rules), then both the non rehab groups i.e. no rehab no book, book only will be stopped and the remaining trial population will be randomised to either the rehabilitation only or rehabilitation and booklet group.

## Discussion

With a sizeable proportion of patients not regaining good function, the outcome of spinal decompression surgery is well short of ideal. This is mirrored in the discectomy population, where many patients are not able to return to work or even fully regain activities of daily living following surgery. The proposed trial will test whether functional outcome following two common spinal operations can be improved via a programme of post-operative rehabilitation that combines professional support and advice with graded, but eventually intense, active exercise or an educational booklet based on evidence-based messages and advice.

## Competing interests

The authors declare that they have no competing interests.

## Authors' contributions

AMcG: Evolved trial, research questions, design and outcomes. CJD: Advised on the trial design, and statistical analysis. TPM: Trial Statistician and co-ordinator of statistical analysis. SM: Trial economist responsible for design of health economics. KJ: Worked with AMcG on concept, trial design, outcome measures and literature reviews. All authors read and approved the final manuscript.

## Pre-publication history

The pre-publication history for this paper can be accessed here:

http://www.biomedcentral.com/1471-2474/11/17/prepub
